# Does ultrasound evaluation of the axilla increase the rate of axillary lymph node dissection in early stage clinically node negative breast cancer patients?

**DOI:** 10.1186/s12893-022-01530-1

**Published:** 2022-03-03

**Authors:** Mahtab Vasigh, Seyed Mostafa Meshkati Yazd, Mohammadreza Karoobi, Reza Hajebi, Adel Yazdankhah Kenari

**Affiliations:** Department of General Surgery, Sina Hospital, Tehran University of Medical Sciences, 14176-13151 Tehran, Iran

**Keywords:** Axilla, Early-stage breast cancer, Ultrasonic evaluation of the axilla

## Abstract

**Background:**

Management and axillary staging of breast cancer has become less invasive and more conservative, over the decades. Considering Z011, axillary lymph node dissection (ALND) can be avoided in T1-2 N0-1 breast cancers with one or two positive sentinel lymph nodes (SLNs), if they are candidates for breast conserving surgery and radiotherapy. The aim of this study was to recognize if pre-operative axillary US evaluation in early-stage breast cancer could lead to more ALND in post Z011 era.

**Method:**

463 breast cancer patients were evaluated. 368 early-stage breast cancer patients (T1-2 N0) were included. We did not perform axillary US in early stage clinically node negative patients; however, 97 patients had axillary US prior to our visit. If axillary US could detect more than two suspicious LNs, US guided biopsy was performed. The remaining clinically node negative patients underwent upfront SLNB. ALND was performed if more than two SLNs were metastatic, or US-guided ALN biopsy proved metastatic involvement.

**Results:**

97 patients had axillary US evaluation before the surgery. 67 patients (69.2%) did not have any suspicious US detected axillary LNs, 17 patients (17.5%) had one, 7 patients (7.2%) had two, and 6 patients (6.2%) had more than two suspicious LNs according to their axillary US evaluation. Those with more than two suspicious LNs underwent ALN US-guided biopsy. Metastatic involvement of the LNs was proved in all of them and they underwent upfront ALND. ALND revealed more than 2 metastatic LNs in 2/6 patients (33.3%). 91 patients who were evaluated by axillary US, had less than two US detected suspicious LNs and underwent SLNB. Amongst 24 patients with one or two US detected suspicious LNs, 1/24 patient had more than two positive SLNs and underwent ALND. In this group 15.6% underwent ALND and 5.2% of them were unnecessary according to the recent guidelines. Axillary US had a false positive rate of 36.6%. The sensitivity of axillary US in distinguishing patients with more than two suspicious LNs in clinically node negative patients was 25%. In the second group (without pre-operative axillary US evaluation), SLNB was performed. 204/272 patients (75%) did not have LN metastasis. 54/272 patients (19.9%) had one or two metastatic SLNs and according to Z011, ALND was omitted. 5.1% had more than two metastatic SLNs and underwent ALND.

**Conclusion:**

US evaluation of the axilla in early stage, clinically node negative breast cancer patients, is not sensitive enough to recognize more than two metastatic ALNs. It leads to more unnecessary ALND. Despite the small number of patients in this study, these results question the rationale of axillary US guided biopsy in low burden (less than two) suspicious LNs. looking for an imaging modality with a higher sensitivity in detecting the Burdon of axillary metastatic involvement is mandatory.

## Introduction

Over the decades, there has been an evolution in the management and axillary staging of breast cancer. It has become less invasive and more conservative, from complete axillary lymph node dissection (ALND) to sentinel lymph node biopsy (SLNB). This strategy avoids potential morbidities of unnecessary ALND such as seroma, pain, neuropathy, limited arm abduction, lymphedema, and increased risk of cellulitis [1]. A decade ago, all patients with a positive metastatic lymph node (LN), either by SLNB or biopsy received a complete ALND [2]. A randomized controlled trial was performed by Oncology Group (Z0011 trial) to determine the effects of ALND after SLNB versus SLNB alone on both survival and 10-years disease free survival. Breast cancer women with primary tumors less than 5 cm, no palpable axillary adenopathy, and 1 or 2 metastatic sentinel lymph nodes were investigated. In a 10-year of follow-up, overall survival for patients treated with sentinel lymph node dissection was not inferior to overall survival for those treated with axillary lymph node dissection [3]. Conclusively, criteria were formulated to describe a sub-group of patients in whom ALND could be omitted without a negative impact on the prognosis [4]. The majority of the studies evaluating the safety of omission of ALND were based on retrospectively collected data, except for the recently published European Organization for Research and Treatment of Cancer (EORTC) AMAROS trial; which states that radiotherapy can be the only axillary treatment in patients with a positive SLN [5]. The results of the Z0011 and AMAROS trial have changed the management of the axilla in early breast cancer. The National Comprehensive Cancer Network (NCCN) even incorporated the Z0011 criteria in their guidelines [6].

Many studies demonstrated the usefulness of preoperative ultrasound (US) evaluation of the axilla. Positive US-guided biopsy of the axillary node was considered a fast track to ALND. In the post Z011 era, a positive axillary node needle biopsy does not indicate an upfront ALND in all patients [7]. Today, it is recommended in some studies that US-guided biopsy is beneficial to patients with multiple suspicious nodes on US [8, 9].

There are reports that go beyond it and question the value of US evaluation of the axilla in patients meeting Z0011 criteria. It is shown that US can’t determine whether more than two LNs are involved [10]. Based on these results some centers changed their clinical guidelines as they do not perform preoperative US of the axilla in clinically T1N0 breast cancer patients any more [11].

In this study, we evaluated the results of axillary management in early-stage breast cancer patients who had the Z011 criteria. They were evaluated in two groups. A group of them had axillary US evaluation pre-operatively and the next group did not. The aim of this study was to recognize if axillary US evaluation could lead to more unnecessary ALND in the patients who met the Z011 criteria.

## Methods and materials

### Patient selection

This retrospective cohort included 463 consecutive patients with invasive breast cancer, in Sina hospital, Tehran, Iran, between 2012 and 2019. The study protocol was approved by the ethics committee of Tehran University of Medical Sciences. Patients with any prior malignancy, inflammatory breast cancer, unknown stage or clinical nodal status, unknown receptor status, or T3 and clinical N1 were excluded. The patients in whom neo-adjuvant chemotherapy (NACT) was the upfront treatment were excluded, too. 368 patients met the criteria to be included in this study. Patients’ data including, age, tumor characteristics, clinical axillary status, results of SLNB and ALND and final pathology reports were abstracted from the medical records. All the patients underwent breast conserving surgery and radiotherapy.

Axillary US evaluation was performed in 97 patients prior to the operation. The radiologist determined, the probability of metastatic involvement of the LN, according to the changes in lymph node shape, fatty hilum status, cortical thickness, and cortical echogenicity. US guided biopsy was performed in patients with more than two US detected suspicious LNs.

SLNB was performed in all the patients without axillary US evaluation or less than 2 US detected suspicious LNs. ALND was performed if more than two metastatic SLNs were recognized intra-operatively, or axillary US-guided biopsy revealed metastatic involvement.

In this study we made a comparison between the clinically node negative patients who were evaluated by axillary US prior to the surgery, with the clinically node negative patients who were not evaluated by axillary US prior to surgery, to illustrate the rate of unnecessary ALND.

### Statistical analysis

SPSS version 20.0 (SPSS Inc., Chicago, IL) was used for statistical analysis. Descriptive analyses compared patient characteristics in two groups. Recorded data were presented as mean with standard deviation (SD) and number (%). The student t test and chi-square were used to compare the baseline continuous and non-continuous variables between two groups, respectively. The level of significance was defined as P < 0.05.

## Results

463 breast cancer patients were evaluated between 2012 and 2019. 369 early-stage (T1-2 clinically node negative cN0) breast cancer patients were included in the final analysis. The mean age of the patients was 44.6 ± 10.3 years old. The most common type of cancer was invasive ductal carcinoma. Table 1 summarizes the characteristics of breast cancer in this study and between two groups.

**Table 1:** Characteristics of the breast tumor among the two groups with and without pre-op axillary US

Characteristics	Non-US group	US group	Total	P.value
Age	44.5 ± 10.85	44.8 ± 9.81	44.6 ± 10.3	0.76
Histologic subtype				0.076
IDC	172 (64.7%)	67 (65.1%)	238 (64.5%)	
ILC	15 (5.6%)	3 (2.9%)	18 (4.9%)	
Medullary	5 (1.9%)	2 (1.9%)	7 (1.9%)	
Metaplastic	2 (0.8%)	5 (4.9%)	7 (1.9%)	
Mucinous	3 (1.1%)	0	3 (0.8%)	
Papillary	2 (0.8%)	3 (2.9%)	5 (1.4%)	
DCIS with microinvasion	7 (2.6%)	2 (1.9%)	9 (2.4%)	
Adenoid Cystic	1 (0.4%)	2 (1.9%)	3 (0.8%)	
No residue (after vacuum biopsy)	7 (2.6%)	2 (1.9%)	9 (2.4%)	
Missing	52 (19.5%)	17 (16.5%)	69 (18.7%)	
Pathologic size of the tumor (mm)	21.44 ± 11.39	21.49 ± 11.05	21.38 ± 11.23	0.81
Grade				
I	39 (14.7%)	14 (13.6%)	53 (14.4%)	0.96
II	96 (36.1%)	37 (35.9%)	133 (36%)	
III	45 (16.9%)	16 (15.5%)	61 (16.5%)	
Missing	86 (32.3%)	36 (35%)	122 (33.1%)	

A correlation between the number of metastatic LNs and Ki67 (P.value: 0.000), tumor histologic type (P.value: 0.000), and unifocality of the tumor (P.value: 0.000) could be observed in this study. The number of metastatic LNs was higher in the tumors located in upper outer quadrant of the breast; however, it was not statistically significant (P.value: 0.058). The number of metastatic LNs increased in higher grade tumors, but it was not statistically significant (P.value: 0.056). We could not find any correlation between the number of metastatic LNs and Her2 status or the size of the tumor.

97 patients had axillary US evaluation prior to the surgery. 67 patients (69.2%) did not have any suspicious US detected LNs, 17 patients (17.5%) had one, 7 patients (7.2%) had two, and 6 patients (6.2%) had more than two US detected suspicious ALNs. US-guided biopsy of the ALN was not performed in the patients who had one or two US detected suspicious ALNs. Those with more than two suspicious LNs underwent US guided biopsy of the LN. Biopsy proved metastatic involvement of the LNs in all of them and they underwent upfront ALND. ALND revealed more than 2 metastatic LNs in 2/6 patients (33.3%).

91 patients with less than two US detected suspicious LNs, underwent SLNB. Only one of them (4.1%) had more than two positive SLNs and underwent ALND. Table 2 summarizes the correlation between axillary US findings and the final SLNB or ALND pathologic results. Axillary US had a false positive rate of 36.6%. The sensitivity of axillary US in distinguishing patients with more than two suspicious LNs in clinically node negative patients was 25%.

**Table 2:** Correlation of US findings and SLNB or ALND pathologic results

SLNB findings	0 metastatic SLN	1 metastatic SLN	2 metastatic SLN	More than 2 and ALND performed	Missing	Total
US suspicious LNs number						
Missing	–	–	–	–	–	–
0 LN	39 (58.2%)	11 (16.4%)	3 (4.5%)	5 (7.5%)	9	67 (69.1%)
1 LN	7 (41.2%)	1 (5.9%)	0	1 (5.9%)	8	17 (17.5%)
2 LNs	4 (57.1%)	0	0	0	3	7 (7.2%)
More than 2 and upfront ALND performed	0	2 (33.3%)	2 (33.3%)	2 (33.3%)	0	6 (6.2%)

SLNB was performed in 272 clinically node negative patients who were not evaluated by axillary US pre-operatively. 204/272 patients (75%) did not have LN metastasis. 54/272 patients (19.9%) had one or two metastatic SLNs and according to Z011, ALND was omitted. 5.1% had more than two metastatic SLNs and underwent ALND. Table 3 summarizes the pathologic results of SLNB in the patients who were not evaluated by axillary US, pre-operatively.

**Table 3:** Number of positive SLNs in 272 clinically node negative patients without pre-operative axillary US evaluation

Positive SLNs	Number	Percent (%)
0	204	75
1	39	14.3
2	15	5.5
More than 2 and ALND performed	14	5.1
Total	272	100

Overall, 363 patients were evaluated by SLNB. 73.8% did not have any positive SLNs, 15.1% had one positive SLN, 5.3% had two positive SLNs and 5.9% had more than two positive SLNs and underwent ALND. ACOSOG Z0011 could prevent ALND in 20.4% of the patients in this study. We could find a higher rate of ALND with axillary US evaluation of the axilla pre-operatively (15.5% vs 5.1%).

## Discussion

We did not perform US-guided biopsy in low burden (less than two suspicious LNs) US detected axillary involvement. Accordingly, the likelihood of proceeding directly to ALND as a result of positive US-guided biopsy was 6.1%, which is three times as low as in general breast cancer population [12]. IT is illustrated in previous studies that pathologic tumor size, and tumor location within the breast are predictive factors for lymph node metastases [13]. Meanwhile in this study we could not illustrate a correlation between the tumor size and location of the tumor, with ALN metastatic burden (P.value: 0.058).

30/97 patients (30.9%) had US detected suspicious ALNs. 23/30 patients (76.7%) had negative or less than 2 positive SLNs and were spared the morbidity of ALND. Our findings were compatible with other studies [8]. If a physician wants to apply Z0011 criteria to omit ALND, preoperative clinical and imaging evaluation of the axilla should be able to accurately distinguish patients with 3 or more positive nodes. There is no pre-operative axillary imaging modalities that can accurately differentiate between minimal nodal disease (1–2 metastatic nodes) and a greater burden of nodal disease [14]. US guided biopsy is valuable to patients with multiple suspicious nodes. SLNB without US guided biopsy is suggested if only one abnormal LN is detected on US in the post-Z0011 era [15]. In this study, the sensitivity of axillary US in detecting greater burden nodal disease (more than two metastatic LNs) was 25%. 6/97 (6.1%) of the patients had more than two US detected suspicious LNs and underwent a US biopsy. US biopsy proved to be metastatic in all of them; consequently, proceeded directly to ALND. However, the final pathology revealed less than two metastatic LNs in 4/6 patients (66.6%). Caudle et al. showed that 52% (99/190) of patients whose US-guided biopsy revealed metastasis had 2 or less positive lymph nodes identified in their ALND [16]. Another report from the Mayo Clinic indicated that 48.4% of patients who had axillary nodal metastases identified by US had 2 or less metastatic nodes in ALND [17]. Harris et al. revealed that more than half of patients with preoperative positive nodes proven by US guided biopsy had N1 disease. The N1 disease increased to 73% if the tumor size was less than 2 cm and only 1 abnormal LN was distinguished by US. They concluded that such patients, may undergo attempt at SLNB if they have the ACOSOG Z0011 criteria [7]. Yoo et al. showed that nearly half of patients with a preoperative biopsy-proven ALN metastasis had only 1–2 positive LNs on ALND. They concluded that patients with few abnormal ALNs on only one imaging modality, meeting ACOSOG Z0011 criteria, are appropriate for SLNB and omission of ALND can be considered [9].

In this study, 24.7% were recognized to have one or two metastatic LNs by axillary US evaluation. We did not perform US biopsy in patients with one or two US detected suspicious LNs. SLNB revealed more than two metastatic LNs in4.1% of them. 95.9% of the patients with US detected suspicious LNs were spared from ALND by omitting US guided biopsy.

On the other hand, 272 patients without pre-operative axillary US evaluation were included, as well. Among them14/272 (5.1%) patients had more than two metastatic SLNs and underwent ALND. In the first group evaluated by US, 12/77 (15.6%) underwent ALND and it was unnecessary in 4/12 (33.3%) of them. Accordingly, a higher rate of ALND was recognized in the group who were evaluated by axillary US prior to the surgery compared to the group who were not. We could omit axillary dissection in 20.4% of the patients in this study considering the ACOSOG Z011.

There were some limitations in this study. We did not have a follow up to evaluate the regional recurrence rate. Moreover, axillary US evaluation was performed by different radiologists and could lead to heterogeneity. Furthermore, looking for an imaging modality with a higher sensitivity in detecting the Burdon of axillary metastatic involvement is mandatory. Despite the small number of patients, these results question the rationale of axillary US guided biopsy in low burden (less than two) suspicious LNs.

## Conclusion

Pre-operative Us evaluation of the axilla in early-stage breast cancer is not sensitive enough to detect the burden of axillary involvement. It leads to more unnecessary ALND. Accordingly; we do not recommend US evaluation of the axilla in clinically node negative early-stage breast cancer patients who are eligible for Z0011. However; more prospective trials are needed to confirm this recommendation.

## Data Availability

The datasets used and/or analyzed during the current study are available from the corresponding author on reasonable request and with permission of Research Ethics Committee of School of Medicine-Tehran University of Medical Sciences.

## Abbreviations

ALND: Axillary lymph node dissection
SLNB: Sentinel lymph node biopsy
US: Ultrasound
FNA: Fine needle aspiration
ALN: Axillary lymph node
EORTC: European Organization for Research and Treatment of Cancer
NCCN: National Comprehensive Cancer Network
NACT: Neo-adjuvant chemotherapy
FNAB: Fine needle aspiration biopsy
US-FNAB: Ultrasound-guided fine-needle aspiration biopsy
